# Using Pedometers to Promote Physical Activity Among Working Urban Women

**Published:** 2006-03-15

**Authors:** Samantha Garbers, Jennifer A Nelson, Terry Rosenberg, Mary Ann Chiasson

**Affiliations:** Medical and Health Research Association of New York City, Inc, New York, NY; Medical and Health Research Association of New York City, Inc, New York, NY; Medical and Health Research Association of New York City, Inc, New York, NY; Medical and Health Research Association of New York City, Inc, New York, NY

## To the Editor:

In light of recent research showing that the prevalence of overweight and obesity among American men and women has reached 65% (1), scientific and media attention has been increasingly focused on ways to address this epidemic. One approach has been to increase Americans' physical activity levels, because even a moderate increase in physical activity may prevent weight gain (2). Evidence, including the study by Wilson et al recently published in *Preventing Chronic Disease* (3), suggests that walking-based programs, particularly those incorporating pedometers, may be an effective way to increase individuals' physical activity levels (4). As a first step toward implementing a similar program among urban working women — who face numerous barriers to structured physical activity — a project was undertaken with two goals. The first goal was to compare the performance of two pedometer models: a low-cost maternal and child health (*low-cost MCH*), which costs $2.90 and is marketed to maternal child health programs, and a *Consumer Reports* best buy (*CR best buy*), which costs $20.00 and was rated by *Consumer Reports* as a best buy for accuracy, ease of use, and value (5). The second goal was to determine the real-life feasibility of pedometer use among working urban women.

Participants were recruited through workplace-based e-mail and flyer postings at public health project sites of a large public health organization in New York City in March and April 2005. Women of any age who worked full time or part time at participating projects and had no physical limitations that would require the use of special equipment, such as a wheelchair or cane, were eligible to participate in the study. The protocol and data collection materials for the internally funded study were approved by the Medical and Health Research Association's institutional review board. 

Of the 30 women who agreed to participate in the study and completed the baseline survey, two did not provide any information on their pedometer use and were excluded from this analysis (n = 28). Participants were asked to wear two pedometers — one on each hip — every day for 6 days, to record on a preformatted daily log sheet the number of steps measured by each pedometer, and to track any problems with use, such as forgetting, dropping, or losing the pedometers. Participants were told that they did not have to increase their physical activity for the study. The Table summarizes the participants' characteristics.

Half of the participants (n = 14) did not wear the pedometers on at least 1 day; from a total of 168 user-days in the study, pedometers were worn on 133 user-days (79% of all days). The most common reasons for not wearing the pedometers were forgetting (28 user-days), losing the pedometers (4 user-days), and wearing a dress (3 user-days). The low-cost MCH pedometer functioned on only 80 user-days, representing 60% of user-days worn, while the *CR* best-buy pedometer functioned on all days worn. Counts on both pedometers were accidentally reset on approximately 10% of user-days worn. The low-cost MCH pedometer fell off or was dropped more frequently than the *CR* best-buy model (7% versus 2% of user-days worn). In all instances of resetting or dropping, the pedometers continued to measure steps throughout the day. Although the difference between the two models in mean daily step counts for the 6-day study period was only 75 steps, on 45% of the 78 days on which both models were worn and functioning, the daily step counts differed between the two models by more than 2000 steps (Figure). Using measurements from the *CR* best-buy model, we found that fewer than 12% of participants achieved the popularly cited goal of 10,000 steps per day (6), and more than half had fewer than 5000 steps per day. As shown in the Table, women who drive to work took significantly fewer steps per day compared with women who ride the subway, train, or bus, and women who mostly sit or stand at work took significantly fewer steps per day compared with women who mostly walk at work. One-way analysis of variance [ANOVA] was used to compare means.

**Figure:**
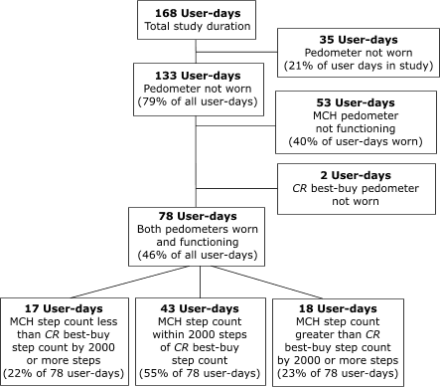
Summary of low-cost maternal and child health (MCH) pedometer model performance compared with *Consumer Reports* (*CR*) best-buy model performance.

**Table:** 

(starting at the top and going left to right)
Box 1 (at the top) reads: 168 User-days Total study duration
A line leads down to Box 3, and another line leads from that line to Box 2, on the right.
Box 2 reads: 35 User-days Pedometer not worn (21% of user days in study)
Box 3 reads: 133 User-days Pedometer not worn (79% of all user-days)
A line leads down from Box 3 to Box 6, and two lines lead from that line to to Box 4 and Box 5 on the right.
Box 4 reads: 53 User-days MCH pedometer not functioning (40% of user-days worn)
Box 5 reads: 2 User-days CR best-buy pedometer not worn
Box 6 reads: 78 User-days Both pedometers worn and functioning (46% of all user-days)
Boxes 7, 8, and 9 are connected to Box 6 with lines.
Box 7 reads: 17 User-days MCH step count less than CR best-buy step count by 2000 or more steps (22% of 78 user-days)
Box 8 reads: MCH step count within 2000 steps of CR best-buy step count (55% of 78 user-days)
Box 9 reads: 18 User-days MCH step count greater than CR best-buy step count by 2000 or more steps (23% of 78 user-days)

In this study, the low-cost MCH pedometer model was found to be largely ineffective. Although the CR best-buy model was more reliable in terms of user-days functioning, developers of walking programs using pedometers need to consider cost in addition to reliability. In this study, the benefit of keeping costs low was offset by the poor functioning of the low-cost MCH pedometer model. Programs should carefully evaluate the reliability of any pedometer before use in an intervention.

This study had the following limitations: all participants were employees of a large public health organization and may have been more aware of physical activity interventions; the data were collected through self-report; and participants were not asked to change their physical activity, unlike participants in an intervention program. This feasibility study revealed some difficulties in the day-to-day use of pedometers, including the pedometers not being worn and accidentally resetting counts. The high proportion of women who forgot to wear the pedometer on at least 1 day during the short study duration of 6 days (50%) suggests that programs using pedometers to promote physical activity must also incorporate motivational techniques and reminders for participants. 

## Figures and Tables

**Table:** Characteristics and Pedometer Use of Participants in a Study to Promote Physical Activity Among Working Urban Women (n = 28)^a^

**Characteristics**	**% of Participants**	**Mean Daily Steps (95% CI)^b^**	***P* Value**

**Age, y**			

≤39	54	5762 (3989-7536)	.47
≥40	46	4812 (2573-7052)	

**Race or ethnicity**			

Latina	43	6548 (4087-9010)	.14
Black, non-Hispanic	29	3451 (1915-4988)	
White, non-Hispanic	21	5080 (493-9666)	
Asian, non-Hispanic^c^	7	—	

**Body mass index**			

Normal (18.5-24.9)	43	5791 (3220-8362)	.10
Overweight (25.0-29.9)	25	6909 (3589-10228)	
Obese (≥30.0)	32	3572 (2235-4909)	

**Children in household**			

At least one child (aged <18 y) in household	57	4740 (3519-5961)	.36
No child in household	43	6005 (3319-8692)	

**Work setting**			

Sedentary (mostly sitting or standing)	82	4548 (3550-5547)	.002
Mostly walking	18	9590 (1761-17419)	

**Usual mode of commuting to work**			

Drive automobile	36	3302 (2000-4605)	.009
Ride train, subway, or bus	64	6587 (4799-8376)	

**Study compliance**			

Wore both pedometers all 6 days^d^	50	5039 (3167->6910)	.64
** **Forgot to wear pedometers at least 1 day	50	5657 (3506-7808)	

a Comparison of means excludes participants who did not wear pedometers on any day. One-way analysis of variance (ANOVA) was used to compare means.

b CI indicates confidence interval

c Asian participants were excluded from comparison of means because of small sample size (n = 2)

d Includes days when pedometers were worn but not functioning; excludes days when pedometer was forgotten, lost, or not worn because participant wore a dress.
